# Effect of Platelet-Rich Plasma on Corneal Epithelial Healing after Phototherapeutic Keratectomy: An Intraindividual Contralateral Randomized Study

**DOI:** 10.1155/2021/5752248

**Published:** 2021-11-27

**Authors:** Kazutaka Kamiya, Masahide Takahashi, Nobuyuki Shoji

**Affiliations:** ^1^Visual Physiology, School of Allied Health Sciences, Kitasato University, Kanagawa, Japan; ^2^Department of Ophthalmology, School of Medicine, Kitasato University, Kanagawa, Japan

## Abstract

**Purpose:**

To assess the effect of platelet-rich plasma (PRP) on the healing response of the corneal epithelium in eyes undergoing phototherapeutic keratectomy (PTK).

**Methods:**

We prospectively examined 20 eyes of 10 patients undergoing bilateral PTK for granular corneal dystrophy or band keratopathy. Patients were randomly assigned to start topical administration of PRP ophthalmic suspension (PRP group) or artificial tears (control group) 4 times daily for 2 weeks. Immediately, 1, and 2 days, and 1 week after PTK, we quantitatively measured the staining area of the corneal epithelium, using slit-lamp photography. We also determined the subjective symptoms and the satisfaction, using the visual analogue system (VAS).

**Results:**

The staining area in the PRP group was significantly smaller than that in the control group on days 1 and 2 (Wilcoxon signed-rank test, *p* = 0.022 and *p* = 0.017, respectively), but not on day 7 (*p* = 0.317). The recovery rate of the corneal epithelium in the PRP group was significantly higher than that in the control group on days 1 and 2 (*p* = 0.022 and *p* = 0.017, respectively), but not on day 7 (*p* = 0.317). We found no significant differences in pain (*p* = 0.139), foreign body sensation (*p* = 0.108), epiphora (*p* = 1.000), or satisfaction (*p* = 0.295), between the two groups. Postoperative complications did not occur in any of the eyes in the study.

**Conclusions:**

The PRP treatment was effective for enhancing corneal epithelial recovery in the early postoperative period, without significant adverse events, in post-PTK-treated eyes, suggesting that it may hold promise as one of the treatment options for treating such postsurgical patients.

## 1. Introduction

Platelet-rich plasma (PRP) has been shown to promote wound-healing responses of biological tissues in a variety of medical fields [1, 2]. In the field of ophthalmology, topical PRP treatment has been used for the treatment of various corneal diseases, such as symptomatic dry eye [3–5], ocular surface syndrome after laser in situ keratomileusis (LASIK) [6–8], persistent corneal epithelial defect [9–12], recurrent corneal erosion [13], dormant corneal ulcers [14–17], acute hydrops for keratoconus [18], and limbal stem cell deficiency [19]. These results suggest that PRP is effective for the treatment of such intractable diseases, because it promotes corneal epithelial healing responses, possibly resulting from the release of various growth factors.

Phototherapeutic keratectomy (PTK) has been recognized as an effective surgical procedure to remove diseased tissue by excimer laser photoablation in eyes having opaque cornea such as granular corneal dystrophy and band keratopathy [20–22], but it always requires the removal of the corneal epithelium to some extent. However, to the best of our knowledge, no intraindividual comparison on the healing responses of the corneal epithelium has so far been conducted. Moreover, the effect of a topical PRP treatment on the corneal epithelium healing has also not been elucidated in post-PTK patients having diseased corneas. It may give us intrinsic insights in the possible role of the PRP treatment in the wound-healing responses of the cornea after PTK surgery, since a prospective intraindividual comparative study can provide more accurate information to compare such subtle differences, based on the fact that the patient age and gender were identical and the condition of the ocular surface and the amount of corneal erosion were closely matched, even when the sample size is relatively small. The goal of the current study is to quantitatively assess the wound-healing responses of the cornea, as well as subjective symptoms and subsequent satisfaction, by the use of topical PRP eye solutions in post-PTK patients.

## 2. Materials and Methods

### 2.1. Study Population

This prospective study enrolled 20 eyes of 10 consecutive patients undergoing bilateral PTK at Kitasato University Hospital. The inclusion criteria were bilateral corneal pathology (granular corneal dystrophy or band keratopathy), no concomitant ocular diseases, and no history of ocular surgery. The exclusion criteria were infection, ocular trauma, sever dry eye requiring permanent occlusion of the lacrimal puncta or temporal punctal plug occlusion, meibomian gland dysfunction, or contact lens wear. The patients were randomly assigned to receive either the PRP treatment (PRP group) or the artificial tear treatment (control group). They were instructed to discontinue the use of all ophthalmic solutions for 2 weeks and to instill the PRP or artificial tears only 6 times daily for 2 weeks in each eye. Antibiotic medications were topically administered 4 times daily for 2 weeks in both groups. The study was approved by the Anzen Mirai Committee of Regenerative Medicine (PB3180099) and followed the tenets of the Declaration of Helsinki. Informed consent for PTK surgery was obtained from all patients after explanation of the nature and possible consequences of the study.

### 2.2. Phototherapeutic Keratectomy

We performed bilateral PTK with the VISX Star S4 excimer laser system (Johnson & Johnson Vision, Santa Ana, US) by one experienced surgeon (KK) and used the following parameters: wavelength, 193 nm; fluency, 160 mJ/cm^2^; repetition rate, 10 Hz; ablation zone diameter, 6.5 mm (including transition zone, 0.5 mm); and ablation depth, approximately 45 *μ*m for granular corneal dystrophy and 35 *μ*m for band keratopathy, based on the depth of pathology using an anterior segment optical coherence tomography (CASIA 2™, Tomey, Aichi, Japan). We used the transepithelial technique for the removal of the corneal epithelium (in a depth of 50 *μ*m).

### 2.3. Preparation of Autologous Platelet-Rich Plasma Eye Drops

The PRP preparation was made following strict sterility conditions, with sterile and disposable materials (TriCell PRP Kit, REV-MED Inc, South Korea) and operating inside a sterile hood bench. Approximately 30 to 32 ml of the patient's whole blood was extracted into a syringe containing 3 ml of ACD-A as anticoagulant. The collected blood was injected into a TriCell PRP Kit in the clean bench and centrifuged at 3,200 rpm for 4 minutes. Upon confirming the existence of buffy coat, the kit was gently swirled to mix plasma and buffy coat. The final plasma quantity level was set between 1 and 5 ml, and a second centrifugation was performed at 3,300 rpm for 3 minutes. Platelet-poor plasma and PRP were separated, and only PRP was harvested into a syringe. Visual and contamination inspections were conducted for the PRP. The PRP in the syringe was then injected into a sterile eye drop bottle labeled with the patient's name and ID and was sealed in a sterile plastic bag.

### 2.4. Clinical Evaluation of Corneal Epithelial Healing

On 0 (immediately after surgery), 1 (24 hours), 2 (48 hours), and 7 days postoperatively, we quantitatively assessed the size of the corneal epithelial defects that were recorded with a cobalt blue filter following instillation of 2 *μ*l 1% fluorescein solution using a slit-lamp microscopy by the same magnification. We analyzed the recorded images by using the ImageJ software (ImageJ 1.48, National Institute of Mental Health; http://rsb.info.nih.gov/ij/) by the binary process. The total size of the corneal epithelial defects was calculated and expressed as that relative to the whole area.

### 2.5. Statistical Analysis

We conducted statistical analyses by using a commercially available statistical software (Bell Curve for Excel, Social Survey Research Information Co, Ltd., Tokyo, Japan). The Wilcoxon signed-rank test was used for statistical analysis to compare the pre- and posttreated data. The results are expressed as mean ± standard deviation (SD), and a value of *p* < 0.05 was considered statistically significant.

## 3. Results

### 3.1. Study Population

The demographic data of the study population are summarized in Table 1. We found no intraoperative complications in the two groups.

### 3.2. Corneal Epithelial Healing

**Figure 1:**
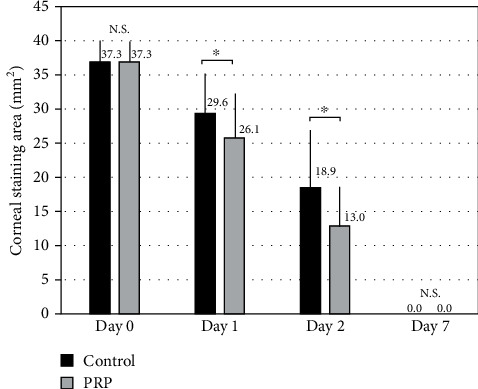
Time course of corneal staining area after topical PRP treatment. We found significant differences on days 1 and 2 (Wilcoxon signed-rank test, *p* = 0.022 and *p* = 0.017, respectively), but not on day 7 (*p* = 0.317).

**Figure 2:**
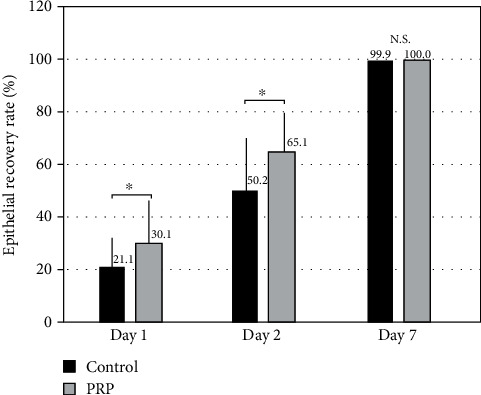
Time course of the recovery rate of the corneal epithelium after topical PRP treatment. We found significant differences on days 1 and 2, but not on day 7, between the two groups. The recovery rate was determined as corneal staining area on day 0 minus that on day *X* divided by corneal staining area on day 0.

The staining area in the PRP group was significantly smaller than that in the control group on day 1 and day 2 (Wilcoxon signed-rank test, *p* = 0.022 and *p* = 0.017, respectively), but not on day 7 (*p* = 0.317) (Figure 1). The recovery rate of the corneal epithelium in the PRK group was significantly higher than that in the control group on day 1 and day 2, but not on day 7 (Figure 2).

### 3.3. Subjective Symptoms and Satisfaction

**Figure 3:**
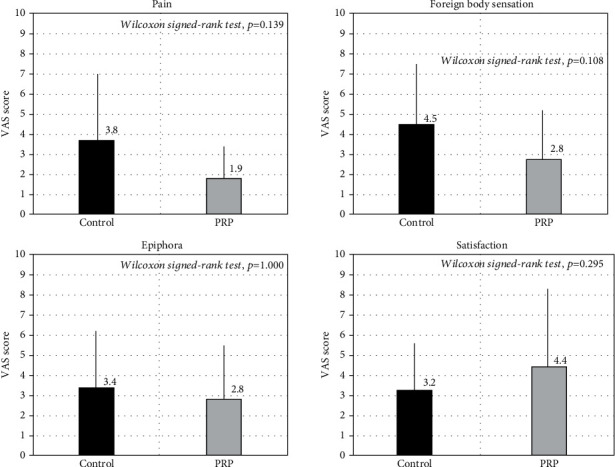
Subjective symptoms and satisfaction after topical PRP treatment. We found no significant differences (pain *p* = 0.139, foreign body sensation *p* = 0.108, epiphora *p* = 1.000, and satisfaction *p* = 0.295).

We found no significant differences in pain (*p* = 0.139), foreign body sensation (*p* = 0.108), epiphora (*p* = 1.000), or satisfaction (*p* = 0.295), between the two groups (Figure 3).

### 3.4. Adverse Events/Complications

We found no significant complications after both treatments, such as persistent corneal erosion, ocular inflammation, persistent ocular pain, infection, or vision-threatening complications throughout the postoperative follow-up period, in the two groups.

## 4. Discussion

In the current study, our results showed that topical PRP treatment was effective for promoting corneal epithelial recovery in the early postoperative period and that no significant complications did occur at any time during the observation period. As far as we can ascertain, this is the first intraindividual contralateral randomized study to assess corneal epithelial healing after instillation of PRP solutions in patients undergoing PTK. Until now, topical PRP treatment has been reported to be effective for the treatment of several ocular surface disorders, such as symptomatic dry eye [3–5], ocular surface syndrome after laser in situ keratomileusis (LASIK) [6–8], persistent corneal epithelial defect [9–12], recurrent corneal erosion [13], dormant corneal ulcers [14–17], acute hydrops for keratoconus [18], and limbal stem cell deficiency [19]. Although there have so far been several studies on the effect of topical PRP solutions on corneal epithelial healing, this intraindividual study indicated that the PRP was safe and effective for promoting corneal epithelial recovery and that it reduced subjective symptoms and increased patient satisfaction, in post-PTK patients.

Platelets deliver a lot of growth factors, cell adhesion molecules, and cytokines from alpha granules. The released growth factors prompt a cascade of various reactions responsible for migration, mitosis, extracellular matrix formation, and angiogenesis promoting proliferation and differentiation of corneal epithelial cells [23]. Although the exact mechanism of the PRP treatment for such ocular surface disorders still remains unanswered, the presence of various growth factors, such as platelet-derived growth factor (PDGF) and epithelial growth factor (EGF), plays a vital role in promoting proliferation and differentiation of corneal cells [9, 24]. Kim et al. described that topical PRP was effective for the treatment of persistent epithelial defects, possibly due to its high concentration of platelet-contained growth factors, most notably EGF [9]. Rodriguez et al. recently demonstrated that the concentration of PDGF-BB and EGF was significantly higher, when the PRP eye drops were frozen at -20°C [24]. Further basic research is necessary to clarify this point.

There are at least two limitations of this study. One is that the sample size is still limited, since the study design was intraindividual and required bilateral PTK having diseased corneal pathology. We enrolled a small sample size, largely due to the ethical considerations, but the intraindividual study can provide more solid outcomes, as mentioned above, even when the sample size is relatively small. Another limitation is that we assessed the staining area only at 4 time points (0, 1, 2, and 7 days), since a frequent follow-up is still a burden not only for the patients but also for the examiners. Accordingly, we cannot exactly determine the postoperative period to corneal epithelial recovery. Although we actually did not include patients using drugs such as antiglaucoma medications in this study, we assume that the use of some drugs does not affect the current outcomes of corneal epithelial recovery, especially in consideration of such faster wound healing within one week in all patients.

In conclusion, our results revealed that the corneal staining area in the PRP group was significantly smaller than that in the control group on days 1 and 2. In our experience, this treatment modality may contribute to the improvement of corneal epithelial recovery, even in diseased eyes having corneal pathology. A further study with a large cohort of postsurgical patients may be warranted to confirm our preliminary findings.

## Figures and Tables

**Table 1:** Preoperative demographics of the study population.

Mean ± standard deviation (range)	
Number of eyes	20
Age	67.1 ± 11.8 years (53 to 86 years)
Male : female	6 eyes : 14 eyes
LogMAR CDVA	0.28 ± 0.48 (-0.08 to 1.70)
Mean keratometric readings (D)	45.3 ± 1.6 D (42.0 to 48.0 D)
Central corneal thickness (*μ*m)	533 ± 26 *μ*m (488 to 589 *μ*m)
Corneal pathology	Granular corneal dystrophy 10 eyes Band keratopathy 10 eyes

LogMAR: logarithm of the minimal angle of resolution; CDVA: corrected distance visual acuity; *D*: diopter.

## Data Availability

The data that support the findings of this study are available from the corresponding author, KK, upon reasonable request.
